# A case report of bladder leiomyoma: an unusual bladder tumour

**DOI:** 10.1093/jscr/rjac580

**Published:** 2022-12-28

**Authors:** Andi Stanescu, Stephanie F Smith, Richard Ball, Utsav Reddy, Alexios Tsiotras

**Affiliations:** Department of Medicine, Norfolk and Norwich University Hospitals, Colney Lane, Norwich NR4 7UY, UK; Department of Urology, Norfolk and Norwich University Hospitals, Colney Lane, Norwich NR4 7UY, UK; Department of Pathology, Norfolk and Norwich University Hospitals, Colney Lane, Norwich NR4 7UY, UK; Department of Urology, Norfolk and Norwich University Hospitals, Colney Lane, Norwich NR4 7UY, UK; Department of Urology, Norfolk and Norwich University Hospitals, Colney Lane, Norwich NR4 7UY, UK

## Abstract

Leiomyomas are benign mesenchymal tumours that rarely arise in the bladder. We present a case of a 53-year-old female who was incidentally diagnosed with a bladder leiomyoma identified on CT imaging performed for investigation of a urachal remnant. We discuss the investigation and management of this lesion in the context of modern urology practice. An awareness of this unusual tumour is important for urologists, who may encounter similar cases in their general urology practice.

## INTRODUCTION

Bladder leiomyomas represent less than 0.5% of all bladder tumours [1]. These are rare, benign, mesenchymal tumours of the bladder with fewer than 250 cases previously reported [2]. We present a case of a 53-year-old female who was incidentally diagnosed with a bladder leiomyoma identified on CT imaging performed for investigation of a urachal remnant. We discuss the investigation and management of this lesion in the context of modern urology practice. An awareness of this unusual tumour is important for urologists, who may encounter similar cases in their general urology practice.

## CASE REPORT

A 53-year-old female was referred for outpatient urological assessment because of persistent microscopic haematuria, urinary frequency and dysuria. Past-medical history included hypercholesterolemia, gastro-oesophageal reflux disease and haemorrhoidectomy. She was an ex-smoker. There was no relevant urological family history. She had been investigated for storage lower urinary tract symptoms 20 years ago and had had normal flexible cystoscopy and ultrasound.

**Figure 1:**
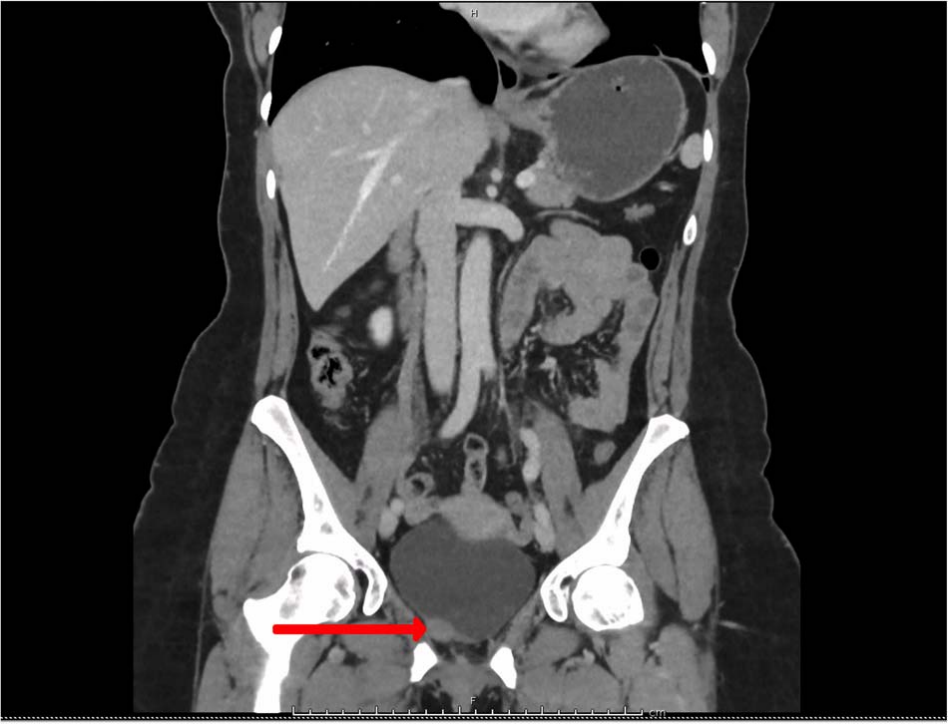
CT urogram (coronal view) revealing a 1.5 cm thickening at the level of the right lateral bladder neck and a 1-cm-soft tissue thickening at the bladder dome.

**Figure 2:**
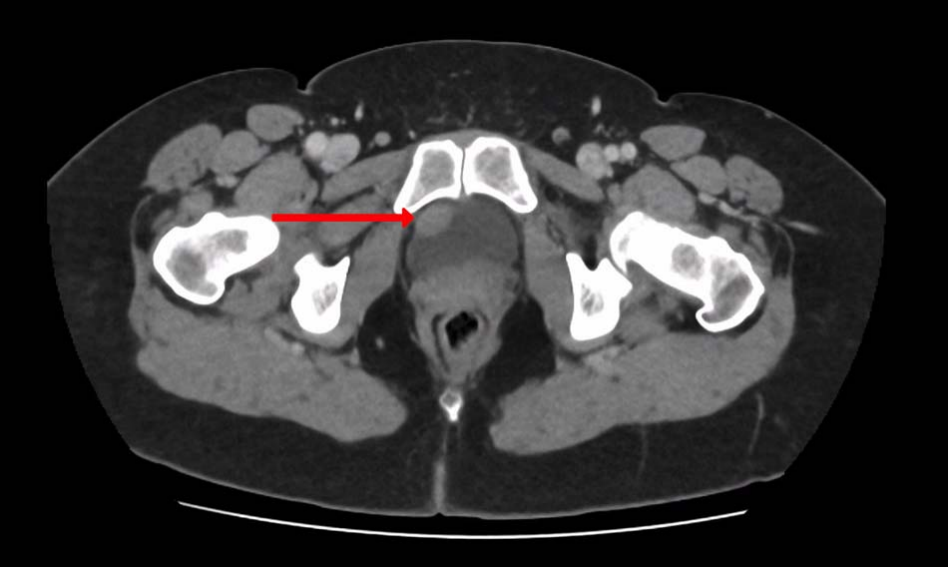
CT urogram (axial view) revealing a 1.5 cm thickening at the level of the right lateral bladder neck, and a 1-cm-soft tissue thickening at the bladder dome.

Blood tests included full blood count revealing all parameters within normal limits, eGFR >90 ml/min/1.73m^2^, Creatinine 64 umol/L and urea 4.5 mmol/L. Urine cultures were negative for infection. Ultrasound revealed a normal-appearing urinary tract, with both kidneys normal in size, shape and echopattern. Flexible cystoscopy revealed only a cystic lesion near the bladder dome which instigated further investigation; subsequent contrast-enhanced Computerized Tomography (CT) abdomen-pelvis scan showed a potential urachal remnant consistent with the flexible cystoscopy finding. In addition, a 1.5-cm-soft tissue structure was identified near the bladder neck which was not clearly seen initially at cystoscopy (Figs 1 and 2).

**Figure 3:**
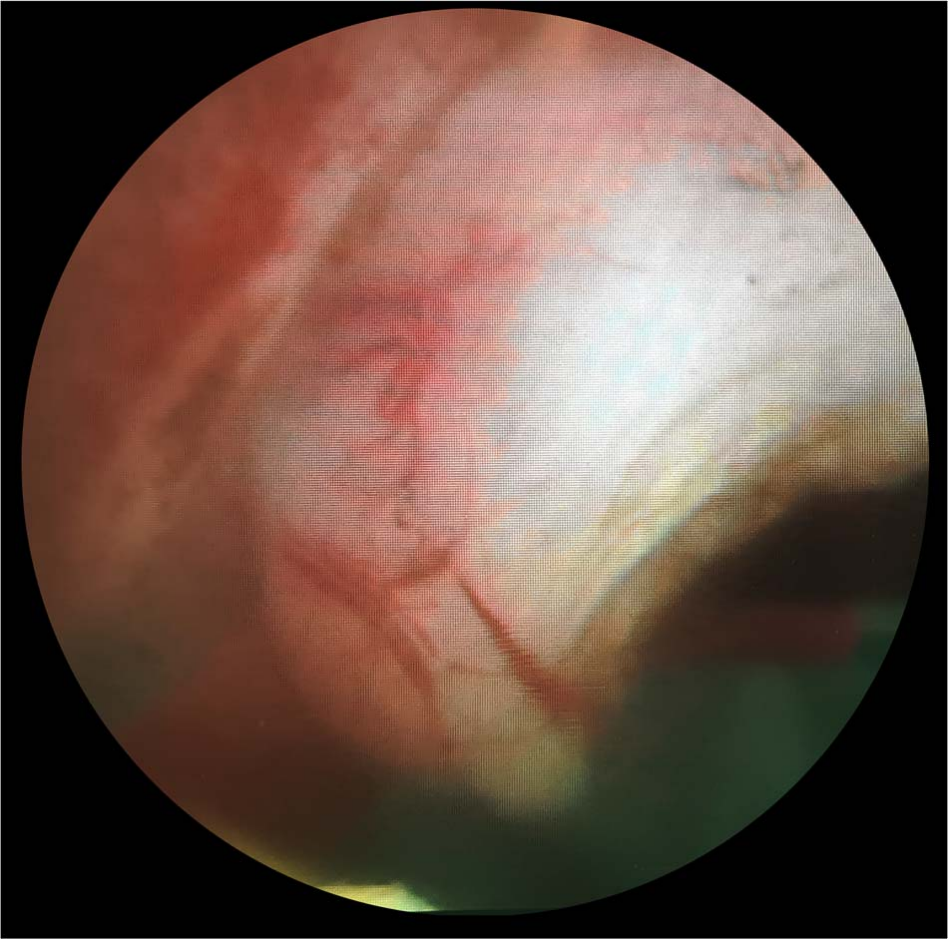
Endoscopic appearances during transurethral resection of the lesion. Note the presence of normal overlying mucosa and the thickened solid-looking non-papillary appearance.

The patient subsequently was booked for diagnostic rigid cystoscopy. On pelvic examination, no palpable mass was felt. Rigid cystoscopy confirmed a cystic structure at the dome that did not look malignant. There was also a mass with normal overlying bladder mucosa on the right side of the bladder neck (Fig. 3), consistent with the soft tissue structure on CT imaging. On resection, the lesion appeared endoscopically as a solid thickened muscular structure, rather than muscle-invasive urothelial cancer.

**Figure 4:**
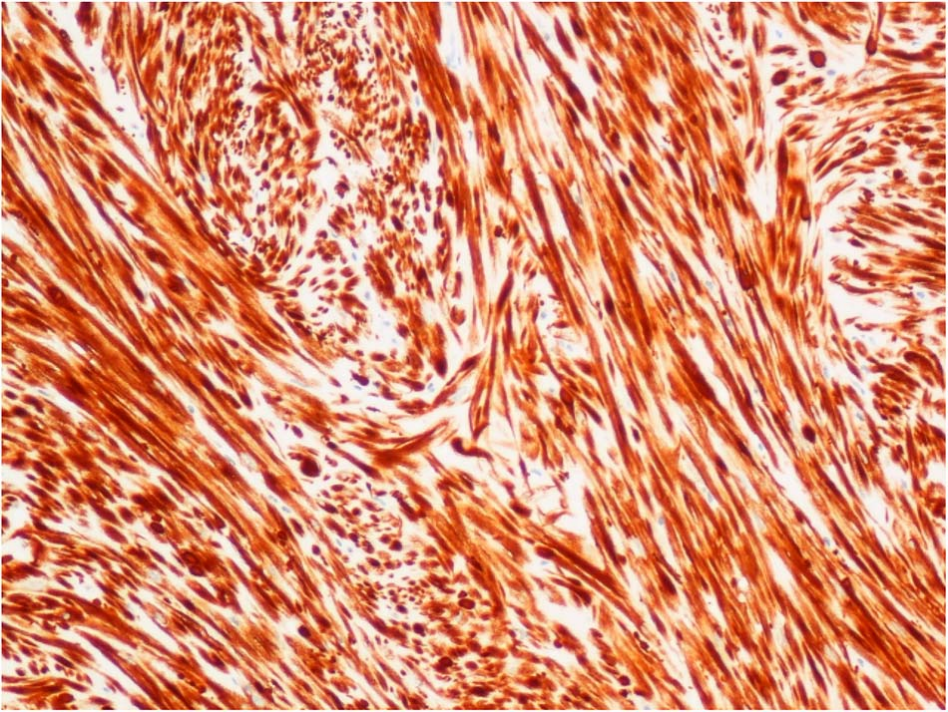
Immunohistochemical staining for desmin, demonstrating the fascicular architecture of bladder leiomyoma (×10 objective).

**Figure 5:**
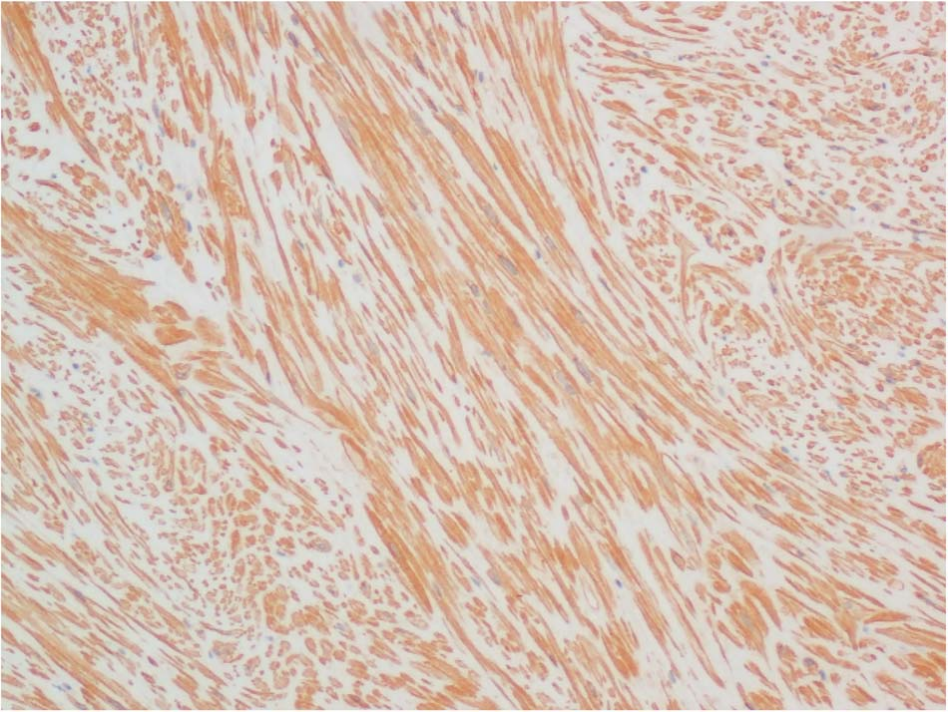
Immunohistochemical staining of an adjacent section for smooth muscle actin (×10 objective).

The histological specimen consisted mainly of muscularis propria of the bladder. Much of it appeared unusually dense and consisted of interwoven fascicles and some storiform areas of bland-looking smooth muscle cells. They were immunoreactive for desmin (Fig. 4) and smooth muscle actin (Fig. 5) but did not stain for ALK 1. In places, there was an associated infiltrate of leukocytes, including groups of lymphocytes, with other areas rich in eosinophils and occasional mast cells. There was no evidence of cytological atypia or malignancy. The appearances were those of a benign leiomyoma.

Her case was reviewed at the specialist multi-disciplinary team meeting, which recommended a bladder MRI scan in 6 months’ time for follow-up.

## DISCUSSION

Although rare, bladder leiomyomas are the most common benign, soft tissue bladder neoplasm [1]. Fewer than 250 cases of bladder leiomyoma have been previously reported [2].

There is a 70% female preponderance, most commonly affecting those in their fourth and fifth decades [3]. An association with female hormones has been postulated [4] and there are histopathological similarities between bladder and uterine leiomyomata.

Symptoms vary according to the location and size of the bladder leiomyoma. With regards to location, they are most commonly endovesical (63%) but also may be intramural (7%) or extravesical (30%) [5]. Intravesical tumours typically present with dysuria, haematuria, urinary urgency (as demonstrated in this case) and occasionally with low back pain, with symptoms being more pronounced in larger tumours. If the tumour has a large pedunculated component that obstructs the bladder neck, this may result in bladder outlet obstruction and subsequent bilateral hydronephrosis [6].

The underlying cause of bladder leiomyoma remains uncertain. However, numerous theories of their pathophysiology have been proposed, summarized by Khater *et al.* [7].

(i) Development is initiated by hormonal influences.
(ii) Dysontogenesis.
(iii) Perivascular inflammation results in metaplastic transformation of bladder vasculature.
(iv) Infection of bladder results in inflammation and tumorigenesis.

The sonographic appearances of leiomyomas consist of a smooth, homogeneous, solid mass, although partially cystic-appearing leiomyomas have been reported [8]. Computed tomography (CT) and magnetic resonance imaging (MRI) can provide more detailed information of the tumour size and location. MRI is the preferred method when looking at the composition and relation to the bladder wall. MRI typically shows an intermediate signal intensity on *T*_1_-weighted images and an intermediate to low signal intensity on *T*_2_-weighted images. After contrast administration, the tumours will show a variable pattern of enhancement, with some enhancing homogeneously and others showing little enhancement [9].

When leiomyomas grow intravesically, cystoscopy (either flexible or rigid) may reveal the presence of a sessile tumour protruding into the bladder lumen. Histopathology from transurethral resection and biopsy is definitive and will exclude malignant tumours (i.e. leiomyosarcoma and muscle-invasive urothelial carcinoma).

It is worth highlighting that the overlying bladder mucosa often appears normal, and therefore, there may be difficulty in diagnosing the abnormality on routine flexible cystoscopy. In this case, the bladder leiomyoma was identified as a result of further investigation of a suspected urachal lesion. We note that this patient had a previous normal flexible cystoscopy some years prior to her presentation.

Bladder leiomyomas can be managed conservatively after diagnosis with transurethral resection and biopsy or can be removed surgically *en bloc*. Asymptomatic patients can be followed up without invasive treatment as to date there are no reported cases of malignant transformation of bladder leiomyomas.

Transurethral resection or *en bloc* surgical removal of bladder leiomyomas may be performed in patients who are symptomatic. The tumour size, extent and location determine the preferred surgical route. The transurethral route is preferred in small intravesical tumours, while a partial cystectomy performed open, laparoscopically or robotic-assisted is indicated for larger intravesical, intramural or extravesical tumours [1, 10].

The prognosis after surgical treatment is favourable and no malignant transformation has been reported to date.

In our case, due to its favourable position and the absence of significant symptoms, transurethral resection was sufficient for diagnosis and the patient is due to be followed up with an MRI scan in 6 months.

## CONFLICT OF INTEREST STATEMENT

None declared.

## FUNDING

None.
